# 

**Published:** 1876-05

**Authors:** 


### ANNOUNCEMENTS FOR THE MONTH.

**MONDAYS.** Societies.
*Mondays, May* 1 and 15—Chicago Med. Society, regular meetings at the Washingtonian Home, 8 P. M.
*Mondays, May* 8 and 22 — Chicago Society of Physicians and Surgeons, regular meetings at Grand Pacific, 8 P. M.
Clinics. *Every Monday.*
At Eye and Ear Infirmary, (Peoria and Adams Sts.) 2 P. M.—Prof. Holmes.
At Central Dispensary (239 W. Van Buren St.), 2 P.M., *Gynecological*—Dr. Adolphus; 3 P.M. *Medical*—Dr. Bridge.
At Mercy Hospital, 2 P. M., *Medical*—Prof. Johnson.

**TUESDAYS.** Societies.
*Tuesday, May* 9—Academy of Sciences, regular meeting, 8 P. M. (263 Wabash Ave).
Clinics. *Every Tuesday.*
At County Hospital, 2 P. M., *Medical*—Prof. Lyman; 3 P. M., *Surgical*—Prof. Freer.
At Mercy Hospital, 2 P. M., *Medical*—Prof. Hollister.

**WEDNESDAYS.** Clinics. *Every Wednesday.*
At County Hospital, 2 P. M., *Ophthalmological*—Dr. Montgomery; 3 P. M., *Gynecological*—Prof. Quine.
At Mercy Hospital, 2 P. M., *Surgical*—Prof. Andrews.
At Central Dispensary, 2 P. M., *Medical*—Dr. Bridge.
At St. Luke's Hospital, commencing at 1.30 P. M., *Surgical*—Dr. Owens.

**THURSDAYS.** Clinics. *Every Thursday.*
At Central Dispensary, 2 P. M., *Diseases of Chest*—Dr. Ingals.
At Mercy Hospital, 2 P. M., *Medical*—Prof. Johnson.

**FRIDAYS.** Societies.
*Friday, May* 12—State Microscopical Society of Illinois, regular meeting at the Academy of Sciences, 8 P. M.
Clinics. *Every Friday.*
At County Hospital, 2 P. M., *Medical*—Prof. Lyman; 3 P. M., *Surgical*—Prof. Freer.
At Mercy Hospital, 2 P. M., *On Diseases of Eye and Ear*—Prof. Jones.
At Central Dispensary, 2 P. M., *Gynecological*—Dr. Adolphus.

**SATURDAYS.** Clinics. *Every Saturday.*
At Rush College, 2 P. M., *Surgical*—Prof. Gunn; 3 P. M., *Diseases of the Brain ana Nervous System*—Dr. Hay.
At Chicago College, 2 P. M., *Surgical*—Prof. Andrews; 3 P. M., *Medical*—Prof. Davis.

☞ At all the above Clinics visiting regular practitioners are, we believe, admitted.

